# Effects of body conformation and udder morphology on milk yield of zebu cows in North region of Cameroon

**DOI:** 10.14202/vetworld.2017.901-905

**Published:** 2017-08-11

**Authors:** Kilekoung Jean-Pierre Mingoas, Julius Awah-Ndukum, Houinga Dakyang, Pagnah André Zoli

**Affiliations:** 1 Department of Physiology and Biochemistry, School of Veterinary Medicine and Sciences, University of Ngaoundere, PO Box 454 Ngaoundere, Cameroon; 2 Department of Anatomy, Histology and Embryology, School of Veterinary Medicine and Sciences, University of Ngaoundere, PO Box 454 Ngaoundere, Cameroon; 3 Department of Biotechnology of Reproduction, School of Veterinary Medicine and Sciences, University of Ngaoundere, PO Box 454 Ngaoundere, Cameroon

**Keywords:** Cameroon, lactation stage, milk yield, teat diameter, parity, zebu cow

## Abstract

**Aim:**

The aim of the study was to assess the effect of udder morphological characteristics on milk yield in zebu cows of Cameroon.

**Materials and Methods:**

The diameter and height of the udder, length and diameter of the teat, and the milk yield were measured in 29 Djafun (Red Mbororo) and 19 Aku (White Fulani) cows in Louggueré zootechnical station in the North region of Cameroon.

**Results:**

Overall, strong positive correlation (r_p_=0.60) between the diameter (240.21±28.58 mm) and height (131.12±23.64 mm) of udders (p<0.001) and between length (39.51±6.44 mm) and diameter (19.85±3.08 mm) of teats (r_p_=0.78) were found in the zebu cows. Udder morphologic characteristics varied significantly (p<0.005) according to breed, lactation stage and parity, and height at whiters. There was significant (p<0.001) correlations between udder diameter (r_p_=0.541) and height (r_p_=0.549) with milk yield.

**Conclusion:**

This study ascertained udder morphological characteristics values in local zebu cows, and showed that udder size is strong and positively correlated to milk yield. The findings are useful in genetic improvement programs of zebu cows.

## Introduction

Milk is important as foodstuff for human consumption and in world food security programs. In Cameroon, milk production was estimated at 56,850 tons in 2012 when 216,000 tons was needed in the country [1]. There is annual individual milk consumption in Cameroon is 14.5 kg, which is less than the recommended amount of 22 kg [2]. Thus, there is gross shortage between milk production and consumption. This situation persists and lower milk production performances of local breeds 0.5-3 L of milk per day are among the contributing factors [1]. Interesting findings have been reported in different species in various areas worldwide to improve milk production. Reports have showed positive correlations between udder characteristics and milk production in Tunisia [3] and India [4,5]. While Ayadi *et al*. [6] reported that daily milk production is positively correlated to distance between teats (r=0.61, p<0.05) and udder depth (r=0.29, p<0.05) in Najdi sheep of Tunisia. In Chile, Daniela [7] recorded positive correlations of 0.77 (p<0.0001) between udder depth and milk production and 0.60 (p<0.0001) between udder height and milk production in local cows. Furthermore, Khan and Khan [8] found genetic and phenotypic correlations between udder biometrics and milk yield in Pakistan Sahiwal cows.

The relationships between udder characteristics and milk yield can be useful tools in selecting animals in dairy production systems. Although there are many livestock species and breeds with good potentials for dairy production in Cameroon, there is a dearth of information on the characteristics of their accessory reproductive organs and how the measurements of these organs relate to reproductive and dairy performances of livestock in the country.

The aim of this study, therefore, was to assess the effect of udder morphological traits on milk yield in local zebu cows in the northern region of Cameroon.

## Materials and Methods

### Ethical approval

Animals were handled and experiments conducted in accordance with laws and regulations of the country and ethical rules (MINEPIA Delegations in North Region; School of Veterinary Medicine and Sciences of the University of Ngaoundere).

### Study location and management of animals

The study was conducted from August to November 2015 in the Louggueré zootechnical station in the northern region of Cameroon (13°30′-13°90′ LE and 9°20′-10°15′ LN).

The animals used in this study were housed on station and had been treated before measurements against intestinal helminthosis and blood parasites with albendazole (Kelaphen^®^, KELA, Belgium) at 7.5 mg/kg orally and diminazene aceturate (Trypadim^®^, MERIAL, France) at 3.5 mg/kg by intramuscular injection following manufacturers’ prescriptions. The animals had also been vaccinated against the prevailing diseases in the region (namely, pasteurellosis, contagious bovine pleuropneumonia, and black quarter) and treated with the acaricide deltamethrin to control ectoparasites. They were grazed on pasture plots on station and given salt every 3 days. Water was available *ad libitum*.

### Udder measurements

The morphological measurements were evaluated on 29 Djafun (Red Mbororo) and 19 Aku (White Fulani) cows with parity and lactation stage varying between 1 and 3 (Table 1), before milking as shown in Table 2 [3,6,7].

**Table-1:** Distribution of study cows according to breed and lactation rank and stage.

Breed	Mbororo Djafun (Red Mbororo) (No. of cows=29)			Mbororo Aku (White Fulani) (No. of cows=19)		
Parity	1	2	3	1	2	3
No. of cows	17	10	2	5	6	8
Lactation stage	1	2	3	1	2	3
No. of cows	9	6	14	7	6	6

Lactation rank (parity): 1=1^st^ lactation, 2=2^nd^ lactation, 3=3^rd^ lactation. Lactation stage: 1=Earlier lactation period, 2=Mid-lactation, 3=Late lactation

**Table-2:** Description of udder morphological measurements.

Udder morphological traits (mm)	Description
Udder depth (Ud)	Distance between udder rear and front attachments
Udder height (Uh)	Distance between cistern abdominal attachment and teat emergence plan
Udder length (Ul)	Distance from the rear attachment of udder to the point where fore udder blends smoothly with the body passing the cloth tape in between right and left teats
Teat diameter (Td)	Measured between rear and front base of teat at emergence of udder

Height at the whiters and thoracic circumference were also measured as previously described by Toszer and Bedo [9].

Milk yield measurement was performed manually once every 2 days, from August to November 2015. After stimulating sucking by calf and proper cow restraint, the overall milk quantity was collected and measured in a graduated container.

### Statistical analysis

Statistical analyses were performed with Fisher least significant differences at p<0.05, using STATGRAPHICS centurion 1/.1.08^®^ software, with the following model:

Y_ijklmn_=μ+T_i_+R_j_+S_k_+P_l_+H_m_+e^ijklmn^, where

Y_ijk_=Studied dependent variable (udder depth, udder height, udder length, teat diameter, and milk production); μ=Mean; T_i_=Genetic effect (i=1-2); R_j_: Effect of lactation rank (j=1-3); S_k_=Effect of lactation stage (k=1-3); P_l_=Effect of thoracic circumference (l=1-19); H_m_: Effect of height at whiters (m=1-20); and e^ijklmn^=Associated random error. Factors such as season of the year and husbandry practices were not considered during analysis.

## Results

### Udder morphological characteristics in zebu cows

The udder morphological traits in lactating zebu cows are presented in Table 3. Overall, the udder mean depth was above 240 mm (maximum of 309 mm and minimum of 143 mm), while mean udder height, length, and teat diameter were more than 131, 39, and 19 mm, respectively. Low values of coefficients of variation were recorded.

**Table-3:** Udder characteristics in zebu lactating cows.

Characteristics	Dimensions (mm)			

	µ±SD	Minimum	Maximum	CV (%)
Udder depth	240.21±28.58	143.00	309.00	11.89
Udder height	131.12±23.64	77.50	197.50	18.02
Teat length	39.51±6.44	22.75	63.50	16.30
Teat diameter	19.85±3.08	12.75	29.00	15.51

µ=Mean, SD=Standard deviation, CV=Coefficient of variation

The udder height was the only morphological characteristic significantly influenced (p<0.001) by udder portion (Table 4). Furthermore, the fore teat mean lengths and diameters were significantly higher (p<0.05) compared to hind teats.

**Table-4:** Variations of morphological characteristics (µ±SD) according to udder portion and cow’s breed.

Variables	Udder portion					

	Left			Right		
Breed	Zebu cows	White Fulani	Red Mbororo	Zebu cows	White Fulani	Red Mbororo
No. of cows	48	19	29	48	19	29
Udder height (mm)	127.24±25.35^A^	135.92±26.89^a^	114.45±15.95^b^	135.03±22.74^B^	142.32±23.93^c^	124.37±15.72^d^
Fore teat length (mm)	41.29±8.04^A^	42.75±8.52^a^	39.16±6.77^b^	40.00±8.77^A^	40.89±10.00^c^	38.69±6.41^d^
Hind teat length (mm)	38.28±8.08^A^	39.65±8.50^a^	36.28±6.97^b^	28.48±7.56^A^	39.69±7.87^c^	38.71±6.55^d^
Fore teat diameter (mm)	20.44±3.82^A^	21.32±4.02^a^	19.16±3.08^b^	20.80±4.23^A^	21.87±4.57^c^	19.25±3.08^d^
Hind teat diameter (mm)	19.04±4.05^A^	19.69±4.21^a^	17.07±3.41^b^	19.11±3.61^A^	20.13±3.70^c^	17.61±2.77^d^

µ=Mean, SD=Standard deviation;

A,B Values with the same superscript in a row are not significantly different (p<0.05);

a,b,c,d Values with the same superscript in a row are not significantly different (p<0.05)

Except for the hind right teat length which did not show any difference according to breed (p>0.05), the udder heights and diameters and teat lengths and diameters, irrespective of the udder portion (left or right), were consistently higher (p<0.05) in the White Fulani than in the Red Mbororo (Table 4).

### Effect of parity and lactation stage on udder morphological traits

**Figure-1:**
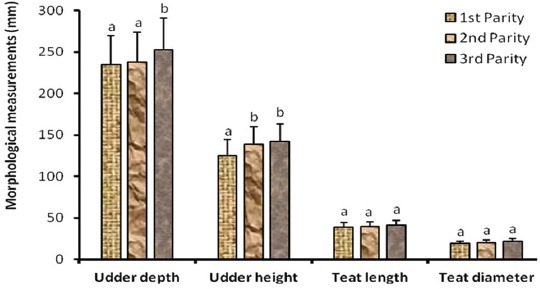
Udder morphological characteristics according to parity in zebu cows.

**Figure-2:**
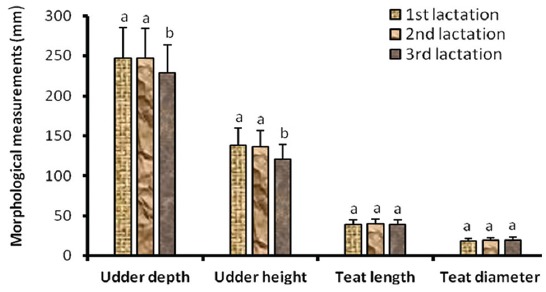
Udder morphological characteristics according to lactation stage in zebu cows.

The results showed that the udder depth significantly increased (p<0.05) at the 3^rd^ parity (Figure-1) and decreased (p<0.05) at the 3^rd^ stage of lactation (Figure-2) while the udder height increased (p<0.05) at the 2^nd^ parity (Figure-1) and decreased at the 3^rd^ stage of lactation (Figure-2).

There was no significant variation (p>0.05) of teat size according to parity and stage of lactation.

### Relation between body conformation, udder morphological characteristics and milk yield

Udder depth, udder height, height at whiters, and thoracic circumference had highly significant (p<0.001) effects on milk yield, while teats characteristics had no effect (p>0.05) (Table 5).

**Table-5:** Variation of milk yield in zebu cows according to morphological characteristics of udder, parity, and lactation stage.

Variables	Production/milking (l)		

	df	CM	p
Breed	1	2.83594***	0.0001
Udder depth	96	0.263927***	0.0000
Udder height	133	0.224538***	0.0007
Teat length	86	0.196823 ns	0.1853
Teat diameter	53	0.153108 ns	0.7783
Parity	2	0.685497*	0.0203
Lactation stage	2	3.10557***	0.0000
Height at whiters	19	1.086***	0.0000
Thoracic circumference	18	1.10232***	0.0000

df=Degree of freedom, CM=Mean square, p=p value, ns=Non significant (p>0.05).

* Significant (p<0.05),

**significant (p<0.01),

*** highly significant (p<0.001)

A positive and highly significant (p<0.001) correlation (r_p_=0.60) was found between udder depth and height (Table 6). The udder size (depth and height) was also strong and positively (p<0.001) correlated (r_p_=0.541 and r_p_=0.549, respectively) to milk yield, especially in White Fulani than in Red Mbororo cows (Table 6). Teat length and diameter were positively and highly (p<0.001) correlated (r_p_=0.78) but were not significantly correlated to milk yield (Table 6).

**Table-6:** Phenotypic correlations (r_p_) between udder morphological characteristics, body conformation and milk yield in zebu cows.

Body conformation	Ud	Uh	Tl	Td	Tc	Hw
Ud	-	0.60***	0.07 ns	0.12 ns	−0.17 ns	0.03 ns
Uh	0.60***	-	0.06 ns	0.13 ns	0.03 ns	0.13*
Tl	0.07 ns	0.06 ns	-	0.78***	0.31***	0.09 ns
Td	0.12*	0.13*	0.78***	-	0.24***	0.12*
Tc	−0.17 ns	0.03 ns	0.31***	0.24***	-	0.31***
Hw	0.03 ns	0.13*	0.09 ns	0.12*	0.31***	-
Milk yield						
Overall zebu cows	0.541***	0.549***	−0.04 ns	0.006 ns	0.12*	0.13*
White Fulani	0.56***	0.57***	−0.21*	−0.19*	0.26**	0.12*
Red Mbororo	0.46***	0.49***	0.17 ns	0.17 ns	0.23*	0.10*

ns=Non significant (p>0.05).

* Significant (p<0.05),

** Significant (p<0.01),

*** Highly significant (p<0.001). Ud=Udder depth, Uh=Udder height, Tl: Teat length, Td=Teat diameter, Tc=Thoracic circumference, Hw=Height at whiters

Body conformation characteristics such as height at the whiters (r_p_=0.13) and thoracic circumference (r_p_=0.12) were moderately correlated to milk yield, especially in White Fulani compared to Red Mbororo cows.

## Discussion

The study showed that overall udder mean depth in zebu cows was 240.21 mm. Udder height, length, and teat diameter means were 131.12 mm, 39.51 mm, and 19.8 5 mm, respectively. Fore teat means lengths and diameters were significantly higher (p< 0.05) compared to hind teats. Udder characteristics varied with breed, udder portion, lactation stage, and parity. Udder size (depth and height) was strong and positively (p<0.001) correlated (r_p_=0.541 and r_p_=0.549, respectively) to milk yield, especially in White Fulani. Low positive but significant correlations were recorded in this study between milk production and height at the whiters (r_p_=0.13) and thoracic circumference (r_p_=0.12).

The udder depth recorded for Mbororo zebu cows in this study is about 1.5 times lower than the 386.8 mm reported for exotic Jersey cows [10]. This may be due to genetic improvement that enhanced udder growth of Jersey cows. The mean teat length and diameter were also lower than the 98.5 and 39.5 mm observed in Brazil Gir cows [11], the 57.7 and 22.73 mm in Simmentale, 53.87 and 21.20 in Holstein in Croatia [12].

Fore teats were more developed than hind teats in the zebus cows contrary to the findings of Daniela [7] and Tina *et* *al*. [12] who reported no significant difference in Holstein and Chile local cows, respectively. These differences are due to morphological traits inherent to Mbororo zebu cows and genetic improvement of Holstein and the local cows in Chile.

Udder measurements such as depth and height, teat length and diameter were significantly higher in White Fulani cows than in Red Mbororo cows. These observations confirm earlier reports of the milk production fitness of White Fulani zebu cows [13].

Udder height and depth increased in second and third parities, respectively. This result agrees with findings reported for Holstein cows that udder height and depth increase with parity [14]. This can be explained by progressive udder hypertrophy with respect to cow’s age and parity [15]. However, this finding is contrary to another report that teats morphology did not varied with parity in Egyptian Buffalo cows [16]. This could be due to genetic characteristics.

Zwertvaegher *et* *al*. [14] reported that in Holstein cows, udder depth and height significantly decreased at third lactation stage, while there were no significant variations of teats morphology with respect to lactation stage. This was associated to involution phenomenon that occurs in udder according to lactation stage [15].

Udder depth and height effects were significantly proportional to milk production, while teat size had no effect on milk yield. Similar findings were reported for local cows in Chile [7], Holstein cows [14] and Simmentale cows [12] who reported that teat size was not correlated to milk production. However, a study on Tinerfen breed goats in Spain [17] stated that udder characteristics related to its globulousness such as volume, perimeter of insertion, and distance between teats are more reliable for milk yield evaluation.

Thoracic circumference and height at the whiters had a significant effect on milk yield in this study. Hans *et al*. [18] found similar results in Holstein cows. Low positive but significant correlations were recorded in this study between height at the whiters, thoracic circumference and milk production, suggesting that relationships exist between body conformation and milk yield in zebu cows. Similar relationships between body conformation and udder morphological development have been reported earlier [19].

In this study, positive and highly significant correlations were observed between udder depth and height and milk yield as earlier reported in Chile local cows [7] and Holstein cows [14]. These correlations were higher in White Fulani compared to the Red Mbororo cows, suggesting that White Fulani cows have a better ability for milk production.

This study, therefore, contributes to better understanding of the udder morphology of Mbororo zebu cows and suggests that large udder depth and height are useful for milk production selection criteria in genetic improvement programs.

## Conclusion

This study ascertained udder morphological characteristics values in local zebu cows and showed that udder size is strong and positively correlated to milk yield. These findings are useful in genetic improvement programs of zebu cows. Further research works should focus on effects of season of the year on udder morphological characteristics and mathematical model building of udder morphology and milk yield correlations in Mbororo zebu cows.

## Authors’ Contributions

HD, KJM, JA, and PAZ conceived, designed and acquired private funding for the study. HD, KJM, and JA coordinated the study design and field work. DH carried out measurements and obtained field data. KJM, HD, JA, and PAZ did the analysis of the information and wrote the manuscript. All authors were involved in revising the manuscript and approved the final manuscript.
